# Supplementary material for: Differential Strain-Specific Responses of Trichoderma spp. in Mycoparasitism, Chitinase Activity, and Volatiles Production Against Moniliophthora spp
Source: Microorganisms. 2025 Jun 27;13(7):1499. doi: 10.3390/microorganisms13071499 (PMC12300225; doi:10.3390/microorganisms13071499)
Supplement: Supplementary file 1 [file microorganisms-13-01499-s001.zip › microorganisms-3649731-supplementary.pdf]

SUPPLEMENTARY MATERIAL

Supplementary Table S1. Metabolic profile of *Trichoderma* spp. and *Moniliophthora* spp. in solo-culturey.

**Strain**	**Retention time**	**Detected compound**	**Percentage area**
M. roreri	35.417	Eicosane	0,74
M. roreri	27.431	n-Hexadecanoic acid	0,23
M. roreri	37.928	Bis(2-ethylhexyl) phthalate	0,97
M. roreri	39.049	Ergosterol	3,07
M. roreri	27.966	Dibutyl phthalate	0,40
M. perniciosa	18.316	Phenol 2,4-bis(1,1-dimethylethyl)-	0,44
M. perniciosa	28.285	n-Hexadecanoic acid	18,41
M. perniciosa	34.718	Heneicosane	1,93
M. perniciosa	37.920	Bis(2-ethylhexyl) phthalate	1,21
M. perniciosa	41.137	Eicosane	1,83
M. perniciosa	27.958	Dibutyl phthalate	0,62
C1 (*T. harzianum*)	26.577	1-Nonadecene	0,26
C1 (*T. harzianum*)	28.523	Hexadecanoic acid	0,55
C1 (*T. harzianum*)	31.071	Eicosane	0,13
C2A (*T. reesei*)	8.154	Phenylethyl Alcohol	0,23
C2A (*T. reesei*)	18.346	Phenol 2,4-bis(1,1-dimethylethyl)-	0,09
C2A (*T. reesei*)	37.972	Bis(2-ethylhexyl) phthalate	0,24
C2A (*T. reesei*)	22.498	Acorenone	0,26
C2A (*T. reesei*)	40.520	Eicosane	0,20
C2A (*T. reesei*)	41.107	1-Nonadecene	0,03
C3A (*Trichoderma* sp.)	18.339	Phenol 2,4-bis(1,1-dimethylethyl)-	0,24
C3A (*Trichoderma* sp.)	27.966	Dibutyl phthalate	0,24
C3A (*Trichoderma* sp.)	28.479	Hexadecanoic acid	14,62
C3A (*Trichoderma* sp.)	37.943	Bis(2-ethylhexyl) phthalate	0,65
C3A (*Trichoderma* sp.)	27.431	Hexadecanoic acid, methyl ester	0,33
C3A (*Trichoderma* sp.)	27.966	Dibutyl phthalate	0,24

C3A (*Trichoderma* sp.)	38.790	Ergosterol	0,40
C3A (*Trichoderma* sp.)	43.752	Lanosterol	0,10
C3B (*T. spirale*)	28.523	Hexadecanoic acid	26,65
C3B (*T. spirale*)	37.935	Bis(2-ethylhexyl) phthalate	0,47
C4A (*T. harzianum*)	28.241	Hexadecanoic acid	2,22
C4A (*T. harzianum*)	37.051	Phenol, 2,4-bis(1-phenylethyl)-	0,61
C4A (*T. harzianum*)	40.520	Eicosane	0,82
C4A (*T. harzianum*)	38.834	Ergosterol	0,13
C4B (*T. glanense*)	28.308	Hexadecanoic acid	11,02
C4B (*T. glanense*)	30.492	1-Nonadecene	0,37
C4B (*T. glanense*)	30.759	Heneicosane	0,11
C5 (*T. spirale*)	18.339	Phenol 2,4-bis(1,1-dimethylethyl)-	0,12
C5 (*T. spirale*)	28.501	Hexadecanoic acid	9,98
C5 (*T. spirale*)	37.958	Bis(2-ethylhexyl) phthalate	0,37
C8 (*T. spirale*)	28.412	Hexadecanoic acid	7,17
C8 (*T. spirale*)	40.513	Eicosane	0,46
C8 (*T. spirale*)	38.752	Ergosterol	0,47
C9 (*T. spirale*)	28.419	Hexadecanoic acid	6,81
C9 (*T. spirale*)	30.506	1-Nonadecene	0,30
C9 (*T. spirale*)	37.512	Eicosane	0,16
C9 (*T. spirale*)	39.042	Ergosterol	1,07
C10 (*T. spirale*)	18.338	Phenol 2,4-bis(1,1-dimethylethyl)-	0,06

Supplementary Table S2. Metabolic profile of *Trichoderma* spp. in dual-culture with *Moniliophthora* spp.

Dual culture	Retention time	Detected compound	Percentage area
M. roreri vs. C1 (T. harzianum)	18,339	Phenol 2,4-bis(1,1-dimethylethyl)-	0,48
M. roreri vs. C1 (T. harzianum)	28,597	n-Hexadecanoic acid	10,67
M. roreri vs. C1 (T. harzianum)	37,958	Bis(2-ethylhexyl) phthalate	0,19
M. roreri vs. C1 (T. harzianum)	27,461	Hexadecanoic acid, methyl ester	1,05
M. roreri vs. C1 (T. harzianum)	25,782	Valencene	0,28
M. perniciosa vs. C1 (*T.* harzianum)	28,634	n-Hexadecanoic acid	8,23
M. perniciosa vs. C1 (*T.* harzianum)	36,004	Eicosane	0,54
M. perniciosa vs. C1 (*T.* harzianum)	39,584	Heneicosane	0,41
M. perniciosa vs. C1 (*T. harzianum*)	27,446	Hexadecanoic acid, methyl ester	0,47
M. roreri vs. C2A (*T. reesei*)	18,338	Phenol 2,4-bis(1,1-dimethylethyl)-	0,38
M. roreri vs. C2A (*T. reesei*)	28,568	n-Hexadecanoic acid	8,42
M. roreri vs. C2A (*T. reesei*)	42.229	Eicosane	0,13
M. roreri vs. C2A (T. reesei)	27,453	Hexadecanoic acid, methyl ester	1,15
M. roreri vs. C2A (T. reesei)	30,774	9-Octadecenoic acid (Z)-, methyl ester	2,04
M. perniciosa vs. C2A (*T. reesei*)	44,859	Eicosane	0,02
M. perniciosa vs. C2A (*T. reesei*)	30,752	9-Octadecenoic acid (Z)-, methyl ester	0,49
M. perniciosa vs. C2A (*T. reesei*)	38,581	Valencene	0,88
M. perniciosa vs. C2A (*T. reesei*)	42,110	α-selinene	0,47
M. perniciosa vs. C3A (*Trichoderma* sp.)	18,353	Phenol 2,4-bis(1,1-dimethylethyl)-	0,06
M. perniciosa vs. C3A (*Trichoderma* sp.)	28,545	n-Hexadecanoic acid	2,39
M. roreri vs. C3B (*T. spirale*)	27,951	Octadecane	1,03
M. roreri vs. C3B (*T. spirale*)	28,538	n-Hexadecanoic acid	6,3
M. roreri vs. C3B (*T. spirale*)	35,439	Eicosane	0,70
M. roreri vs. C3B (*T. spirale*)	35,996	Heneicosane	0,48
M. roreri vs. C3B (*T. spirale*)	37,950	Bis(2-ethylhexyl) phthalate	0,31
M. roreri vs. C3B (*T. spirale*)	27,446	Hexadecanoic acid, methyl ester	0,65

M. roreri vs. C3B (*T. spirale*)	25,774	Valencene	0,29
M. perniciosa vs. C3B (*T. spirale*)	18,339	Phenol 2,4-bis(1,1-dimethylethyl)-	0,79
M. perniciosa vs. C3B (*T. spirale*)	28,479	n-Hexadecanoic acid	8,73
M. perniciosa vs. C3B (*T. spirale*)	35,424	Eicosane	0,74
M. perniciosa vs. C3B (*T. spirale*)	42,066	Heneicosane	0,70
M. perniciosa vs. C3B (*T. spirale*)	27,439	Hexadecanoic acid, methyl ester	0,34
M. roreri vs. C4A (*T. harzianum*)	28,694	n-Hexadecanoic acid	2,55
M. perniciosa vs. C4A (*T. harzianum*)	18,353	Phenol 2,4-bis(1,1-dimethylethyl)-	0,23
M. perniciosa vs. C4A (*T. harzianum*)	28,389	n-Hexadecanoic acid	2,90
M. roreri vs. C4B (*T. ghanense*)	18,309	Phenol 2,4-bis(1,1-dimethylethyl)-	0,62
M. roreri vs. C4B (*T. ghanense*)	30,506	1-Nonadecene	0,52
M. roreri vs. C4B (T. *ghanense*)	35,543	Eicosane	0,44
M. roreri vs. C4B (T. *ghanense*)	35,944	Heneicosane	0,21
M. roreri vs. C4B (T. *ghanense*)	27,431	Hexadecanoic acid, methyl ester	0,55
M. perniciosa vs. C4B (*T. ghanense*)	28,456	n-Hexadecanoic acid	5,07
M. perniciosa vs. C4B (*T. ghanense*)	31,858	Heneicosane	0,69
M. perniciosa vs. C4B (*T. ghanense*)	34,221	1-Nonadecene	0,43
M. perniciosa vs. C4B (*T. glanense*)	39,569	Eicosane	0,48
M. perniciosa vs. C4B (*T. ghanense*)	27.438	Hexadecanoic acid	0,38
M. roreri vs. C5 (*T. spirale*)	28,508	n-Hexadecanoic acid	6,75
M. roreri vs. C5 (*T. spirale*)	28,917	Eicosane	0,79
M. roreri vs. C5 (*T. spirale*)	39,057	Heneicosane	0,33
M. roreri vs. C5 (*T. spirale*)	27,446	Hexadecanoic acid, methyl ester	0,72
M. perniciosa vs. C5 (*T. spirale*)	28,523	n-Hexadecanoic acid	3,06
M. perniciosa vs. C5 (*T. spirale*)	35,439	Eicosane	0,65
M. perniciosa vs. C5 (*T. spirale*)	27,454	Hexadecanoic acid, methyl ester	0,55
M. perniciosa vs. C5 (*T. spirale*)	30,759	9-Octadecenoic acid (Z)-, methyl ester	0,92
M. roreri vs. C8 (*T. spirale*)	27,959	Heneicosane	0,56
M. roreri vs. C8 (*T. spirale*)	28,494	n-Hexadecanoic acid	8,27
M. roreri vs. C8 (*T. spirale*)	39,050	Eicosane	0,56

M. roreri vs. C8 (*T. spirale*)	27,446	Hexadecanoic acid, methyl ester	0,64
M. roreri vs. C8 (*T. spirale*)	25,767	Valencene	0,17
M. perniciosa vs. C8 (*T. spirale*)	28,456	n-Hexadecanoic acid	8,14
M. perniciosa vs. C8 (*T. spirale*)	39,057	Eicosane	0,67
M. perniciosa vs. C8 (*T. spirale*)	39,577	Heneicosane	0,67
M. roreri vs. C9 (*T. spirale*)	18,316	Phenol 2,4-bis(1,1-dimethylethyl)-	0,10
M. roreri vs. C9 (*T. spirale*)	28,411	n-Hexadecanoic acid	11,19
M. roreri vs. C9 (*T. spirale*)	37,928	Diisooctyl phthalate	0,50
M. roreri vs. C9 (*T. spirale*)	39,057	Eicosane	0,25
M. perniciosa vs. C9 (*T. spirale*)	18,301	Phenol 2,4-bis(1,1-dimethylethyl)-	0,12
M. perniciosa vs. C9 (*T. spirale*)	28,441	n-Hexadecanoic acid	11,37
M. perniciosa vs. C9 (*T. spirale*)	35,930	Eicosane	0,44
M. perniciosa vs. C9 (*T. spirale*)	38,790	Ergosterol	0,46
M. perniciosa vs. C9 (*T. spirale*)	30,737	9-Octadecenoic acid (Z)-	0,85
M. roreri vs. C10 (*T. spirale*)	28,568	n-Hexadecanoic acid	10,65
M. roreri vs. C10 (*T. spirale*)	28.902	Eicosane	0,66
M. roreri vs. C10 (*T. spirale*)	42,073	Heneicosane	0,49
M. roreri vs. C10 (*T. spirale*)	27,438	Hexadecanoic acid, methyl ester	0,43
M. roreri vs. C10 (*T. spirale*)	25,760	Valencene	0,30
M. perniciosa vs. C10 (*T. spirale*)	27,268	Octadecane	0,03
M. perniciosa vs. C10 (*T. spirale*)	28,634	n-Hexadecanoic acid	5,30
M. perniciosa vs. C10 (*T. spirale*)	42,400	Eicosane	0,17
M. perniciosa vs. C10 (*T. spirale*)	27,446	Hexadecanoic acid, methyl ester	0,22
